# Participatory research: real or imagined

**DOI:** 10.1007/s00127-018-1549-3

**Published:** 2018-06-21

**Authors:** Diana Rose

**Affiliations:** PO34 Institute of Psychiatry, Psychology and Neuroscience, King’s College London, De Crespigny Park, London, SE5 8AF UK

**Keywords:** User-led research, Participatory research, Mental health

## Abstract

**Background:**

Participatory research has as a central tenet that power relations between researcher and researched be reduced. In the last 20 years, a substantial literature has demonstrated the difficulties inherent in this as well as the troublesome nature of certain central concepts.

**Aims:**

(1) To describe and illustrate a new form of participatory research where the researchers share at least something with the participants in the research. That is, all are users of mental health services. (2) To reflect on the novel form of participatory research in terms of whether it shares, mitigates or avoids some of the difficulties of more traditional forms and to pose the question: what is a mental health community?

**Results:**

The model described is new in that the researchers have a different status than in conventional participatory research. But it is illuminated by and itself illuminates issues of power relations in research and difficulties in reducing that; gatekeepers and the exclusion of crucial groups of service users; the confusion of demographic representativeness with the silencing of marginalized perspectives; coming out of the academic space and the shifting issue of what counts as ‘communities’ in mental health.

**Conclusion:**

The examples given are moderate in scale and relevant to social psychiatry. Yet they may change methods and the definition of participatory research and at the same time be vitiated by but also illuminate dilemmas already identified in the literature albeit in different formations.

## Background

This journal is concerned mostly with large scale epidemiological studies and the importance of, and fascination with, big data is growing. But a complete picture needs to include ecologically valid smaller scale work that is often qualitative. The MRC framework for complex interventions nods in this direction [1] and those concerned with health services as complex systems claim to prioritize it [2–5]. A different method which focuses on qualitative concerns is participatory research, which is the subject of this paper particularly the extent to which it can and does live up to its goal of meaningfully involving communities. This can be on smaller or larger scales and I will use examples of the former but reflect upon more general lessons for methodology and conceptualisation in social psychiatry, particularly.

One of the founding principles of participatory research, and the one of interest here, is that it should level the power relations between researchers and the community in the research itself: in who sets the research agenda, who drives the research process and governs it and who interprets information. In all these aspects of research, the community are no longer ‘subjects’ but equal partners. This at least is the mission but achieving it has often proven difficult [6] and some commentators argue it is often tokenistic sometimes in very subtle ways [7] or can even have paradoxical effects [8].

Even those who largely promote participatory research note that there may be power relations within a community that prevent full participation of all members. Power distributed according to gender is an example and may be hidden if researchers do not reflect on their own gendered positions [9]. This goes beyond the recognition that researchers are unlikely to engage all sections of a community equally because there will always be gatekeepers, often elders in low resource settings [10]. Multiple identities may be at stake intersecting in a complex web of power relations which are very hard to reconcile with the principles of participatory research. Mason and Boutilier [6] describe a project in Canada where academic researchers worked with nurses and it became evident only when reflective workshops were held at the end of the programme that institutional factors had operated to prevent the reduction of power relations which the researchers thought had been accomplished. Maguire [13] argues that participatory research aligns with feminist theory and ways of working and that this can shed light on these significant intersections.

Such complexities can be hidden when large organizations start to promote what their version of ‘participation’ looks like. This criticism was made at an international level when the World Bank began a programme to ensure that ‘communities’ should always participate in certain policy decisions, for example, regarding agriculture [8]. Aside from this being the imposition of an organizational and Western view of participation and ignoring the power relations within communities, Henkel et al. argued that the very concept ‘community’ was then rendered unexamined. This critique has been pushed even further by some to encompass NGOs [11] who claim to represent impoverished or marginalized groups. Most of these arguments are not about participatory research as such (see [12]) but the concerns with a somewhat messianic view of participation combined with an unexamined concept of the ‘community’ regarded as whole and authentic just waiting for expression given the ‘right’ conditions are relevant to the very discrete issue I will address in this paper.

The specific innovation in the work to be described here is that the researchers are more intrinsically part of the community being researched. In this case, all are mental health service users. To my knowledge, this is an innovation which doubles the meaning of ‘participation’. I explain the work by means of an example which is small scale but intensive. The reflections which follow will interrogate the extent of the difference this innovation makes.

### Driving outcome measures from the ground up

The ‘small stage’ version of participatory research that I will describe and then provide critical reflections about has been extensively published mostly in medical journals [12–17] and was developed at a medical/psychiatric institution. The peer review process for the first paper of this kind took 5 years illustrating the unusual nature of the work in such spaces [14].

The ‘participatory’ model at stake here has a rather different claim to reducing power relations than pertains in the ‘industry’ of participatory research but it may not overcome all the difficulties that have been identified in the previous work. I will return to this but for the moment argue that this claim rests on the fact that the researchers are in some sense at least members of the same community as participants. The participants are chosen because they use (or refuse) mental health services and the researchers likewise have used mental health services themselves. This is in contrast to most participatory research projects where researchers are external to the community they work with and have a single ‘professional’ identity though this is constantly put in question. Our double position as both service users and researchers pertained to early work on ‘consumers’ perspectives on electroconvulsive therapy (ECT) where the two main researchers had received ECT themselves. This was part of the reason why results diverged markedly from the conventional literature [18]. However, the main focus of this paper is on a method for using this ‘double identity’ as service user/survivor and researcher to develop something which at first sight looks very conventional: outcome measures. This work has been done mostly in the arena of mental health and the distinctiveness of this is something I shall return to.

Outcome measures in health are generally derived by taking existing scales and attempting to improve on them or by identifying potential scale items through the literature, including the literature on existing measures. Researchers, who often also have a clinical position, take the decisions on what are ‘good’ items guided by conventional psychometric and other statistical techniques. In social science, focus groups of ‘lay’ individuals are often convened but they do not have any special stake in the content of the scales that are being devised and are selected using demographic and market research principles [19]. Again, psychometrics plays a dominant role.

### Patient-reported outcome measures (PROMs)

Patient-reported outcome measures (PROMs) are enjoying a high profile in health research currently in both the UK and USA [20] although it has been argued that this work is atheoretical and that this is responsible for their limited impact [21]. Our critique develops this and rests on the argument that PROMs are limited in impact because they embody a clinical view and ignore the perceptions of patients and service users. In effect, the questions are decided by clinicians and researchers and patients simply fill them out. In this sense, they are indeed ‘patient reported’ but they are not ‘patient devised’. We have, therefore, developed a method for patient-generated patient reported outcome measures (PG-PROMs). The method is described in detail elsewhere [13] but I will develop the salient points here.

To describe this, I will take the example of a PG-PROM developed to discover service users’ perceptions of acute mental health wards [16]. This domain is topical as there is much disquiet about this provision with many seeing such wards as hostile, untherapeutic and unsafe. The measure development started with focus groups but unlike the social science work referenced above, the participants in these groups had been patients in local inpatient wards very recently, within the previous 2 years. They, therefore, had a direct stake in the work undertaken and the significance of their expertise was emphasized at the start. The facilitators were one person with experience of inpatient provision, including involuntary status, and the other also a mental health service user although without inpatient experience. The project also had a reference group mostly made up of people with inpatient experience and this group helped in the construction of the topic guide for the focus groups.

### Focus groups

There were four groups and the aim was originally for one of these to be composed of people with experience of involuntary admission and very likely other coercive practices. This aim was achieved but unsurprisingly in practice all the groups included participants who had been detained and subject to coercive interventions. Forty-nine participants took part in the initial focus groups and I will comment on the demographics later.

Each group met twice and at the first meeting, the facilitators disclosed their mental health experience with the objective of making the groups a non-hierarchical and non-medical space. The groups met in community venues, not hospital premises and food and drink were provided. Participants were paid for their contributions at both groups, although welfare benefit regulations limited the amounts that could be paid. Thus, researchers and researched were not equal in this respect. For the first wave of groups, the topic guide was introduced but during the event participants spoke freely and at length, speaking to the whole of the topic guide and beyond. They did not necessarily cover the domains in the order given but this was deemed irrelevant or even positive as it showed engagement with the process. Many difficult subjects were discussed and although these were often painful there was also a good degree of black humour especially in the group where all had been detained as some knew one another and reminisced about their shared experiences in very lively exchanges.

The group discussions were digitally recorded and transcribed and the researchers analysed the recordings thematically aided by Nvivo 10 software. The aim was to identify and draw out the main themes largely following Braun and Clark [22] but in a provisional way and begin to construct items for the measure. This analysis was brought back to the second meeting of each group to check with them that we had adequately captured what they said [23]. The second meetings also provided an opportunity for participants to add anything they had forgotten. In general, the groups agreed with our analysis of the first meetings and did add a number of things provoked by the initial analysis. At the end of the second meetings, we asked each group for a couple of volunteers to participate in the next stage of the research and said they would all receive a feedback letter when the work was complete. Only one person did not return to the second group showing, I would argue, engagement with the research.

The second group transcriptions were analysed in the same way as the first. Then, from the two sets of analyses the researchers constructed a draft questionnaire. This was done reflexively, drawing on their own experience to help the process as data never speak for themselves. Themes do not ‘emerge’ from transcripts as if they are immanent but require an active process of meaning making and in this case this included reflexive engagement of our own experience as well as the experience of being in the focus groups themselves. This point has been made in the literature [22] but we took it further for a double grounding in the way inpatient wards are perceived by those who use them.

### Expert panels

The next stage of the research consisted of ‘expert panels’ looking at our draft measure and amending it. The first expert panel was made up of the volunteers referred to above, that is, members of the original focus groups. The purpose of this was to check again that the draft measure complied with what the focus groups had intended. The second expert panel was also made up of people who had been inpatients in the local provider Trust in the previous 2 years but this time they were new participants. The aim here was to give an opportunity for fresh eyes to look at the measure. As well as the chance to amend the measure, we also asked the panels to look at the language and layout as previous work had shown some commonly used measures to be either difficult or unattractive to the point of shabbiness [24]. As a result of this exercise, some changes were made to items and language but these did not contest the basic intentions of the original focus groups. There was, therefore, some consensus across participants at least in respect of this particular local provider. Fifteen people took part in the expert panels.

### Feasibility

The final stage of constructing the measure was undertaken not with former service users but with current ones, people who were in psychiatric hospital. We called this the feasibility stage and the aim was to ensure that the measure was easy to complete. We gave it to people in groups of 10. For each group, we examined how they filled it in to identify any ambiguities or difficulties they had. Each time we tried to resolve these ambiguities, we gave it to another group of 10 and so on until we had what appeared to be an unambiguous measure that was easy to complete. This point was reached with the fifth group and this essentially was the end of development of VOICE (Views Of Inpatient CarE), our PG-PROM for service users’ perceptions of inpatient wards.

The aim of this process was to develop a measure grounded entirely in the experiences of mental health service users in contrast to how such measure are usually derived. Presently, I will reflect on some of the difficulties faced and the extent to which this work counts as meaningful participatory research, albeit at this very discrete level. But before this, PG-PROMs go through another stage which although is more in line with conventional measure construction does not dominate the process but comes at the end. That is, we do indeed psychometrically test our measures. We know they have face validity because of the method of generation. We also need to assure that our measures are stable as—results could not be relied on if they were subject to whims of context for example—and so we conduct test–retest reliability procedures with approximately 50 persons.

### Psychometrics

Unlike much psychometric work, ours use both qualitative and quantitative assessment as described by Fitzpatrick et al [3] (http://www.journalslibrary.nihr.ac.uk/hta/volume-2/issue-14). To check on stability, an inter-rater reliability exercise was conducted. In our test–retest check, all participants were current inpatients who completed the measure twice with a 1 week interval between. The inter-rater reliability was very high, much higher than is often the case (Crohnbach’s Alpha 0.9). To standard psychometricians this is curious. Kline [25], in his revised textbook, argues that psychometric testing can only be done where ‘subjects’ are ‘cognitively intact’. Our participants mostly had a diagnosis of active psychosis but they were perfectly able to understand and complete VOICE. I would contend that this was because the measure was user generated and so made sense to them in their current situation and this is an additional argument for conducting psychometrics at the end of a user-focused process rather than giving them methodological prominence.

A second psychometric technique we used was factor analysis, with 137 participants. Although this seems like a complex numerical process, it does in fact require a high measure of intuition [26]. The quantitative output requires interpretation as well as conceptualisation so that factors can be adequately ‘named’. The factor analysis of VOICE resulted in two factors—‘interaction’ and ‘security’. A factor of interaction is not surprising as descriptions of interaction between service users and staff—or, more commonly, the lack of this—are legion in the user literature and often dominated the narrative in the focus groups [27]. Security is a different matter. The factor indicated that feeling safe or not on wards was paramount for the people who used them. On the other hand, we found no factor of the ward environment and this is in contradistinction to most scales. This would seem to be because clinical researchers believe this to be important. Our participants did talk about the state of the physical environment, the food and so on but did not do so at length and so these were not identified as significant themes.

I turn now to consider whether this form of participatory research shares with or avoids the pitfalls identified in the Background. Does having user/survivor researchers meaningfully level power relations and engage ‘communities’. Additional considerations are also raised.

## Reflections

### Participatory research and power

Does the process just described count as participatory research in a meaningful sense? I move now to reflecting on the method given above. First, did we succeed in leveling the power relations between ourselves as user researchers and the participants in the study? At this point, I need to revert to anecdote. We started the focus groups by emphasizing how much their experience was sought to provide a picture of the inpatient experience that had accuracy and depth. On the whole, this together with our own disclosure seemed to make for free and uninhibited discussion. However, one issue arose which suggested that this leveling was not complete. At the end of one focus group, a participant said “oh, I forgot you are not psychiatrists”. For this participant at least, something about the very fact that we ran the groups made them like any other research encounter, and what these participants are used to is research run by clinicians.

The issues raised by authors such as Mason and Boutelier [6] arise in our work as well but differently. Like them, we are not working with a mostly self-contained community but we are working within a distributed structured system—that of psychiatric services in England and in this case a metropolitan area ranking high on every index of deprivation and marginalization. We do not have community gatekeepers but we also do not have unfettered access to potential participants. To find people who would like to participate, our gatekeepers are the mental health professionals that have primary responsibility for them. These are mainly nurses and social workers and they are selective in whom they put forward. They routinely filter out potential participants who they deem too vulnerable or chaotic. They may make reference to ‘lack of capacity’ but this does not mean they have made an assessment under the Mental Capacity Act 2005 (http://www.legislation.gov.uk/ukpga/2005/9/contents); an issue in itself [28]. They may also, for reasons of beneficence, exclude people with ambiguous or no residence rights. In terms of demographics our groups were balanced in terms of gender, ethnicity, age and sexuality but the exclusion of those deemed vulnerable or chaotic or ‘lacking in capacity’ is serious as the experience of inpatient care is likely the most coercive for these groups and yet that is not represented in our measure. This of course is an issue for all research but mostly it figures as a ‘taken for granted’ and not interrogated as to its implications. This is reinforced by its status in formal ethical procedures, a question currently being examined by Lucy Series (pers. comm. January, 2018).

The other way in which power creeps in here is if we are in fact complacent about demographic ‘representativeness’. The researchers in this study were two white, straight women. Research into acute care, such as the study I am discussing, is sometimes conducted with involvement of service user researchers. People from Black and Ethnic Minority communities are vastly overrepresented in this provision, more likely to be detained and compelled, more likely to receive a diagnosis of psychosis, more likely to enter mental health services via the police and less likely to receive psychological services in both the UK [29] and USA [30]. Including BME survivors in such research is vital but at the same time they cannot be expected to carry the weight of the history that has led to this situation. In addition, the anglophone scientific paradigm of generating knowledge through empirical research may not be adequate to capturing the systemic injustices at stake here. In one consultation with BME service users, we repeated a forum for gathering community priorities for research [31]. One conversation focused on turning the tables in research. “Stop treating us as guinea pigs, as the problem. Research yourselves and the systems you create and inhabit”. Making sure samples are ‘representative’ will never touch and certainly will never solve this issue. Perspectives are not reducible to metrics.

### Ethics

It used to be the case that Research Ethics Committees (RECs) were suspicious of user involvement in research for much the same reason as clinicians which is hardly a surprise as clinical views are listened to in RECs. This issue is not as difficult now but the issue of ethical research goes beyond Ethics Committees [32]. We encountered two ethical dilemmas, one serious and organizational and one at first sight personal but with implications for research integrity.

The first concerned treatment on a particular ward that amounted almost to abuse. We had been told about this in the confidential setting of a research group, and so at first thought that formal ethical criteria precluded us from taking it further. However, the situation seemed sufficiently serious to do something about it. We asked our informants for their permission to take it to the Chief Executive of the Trust and they readily agreed. As this individual was familiar to the research team, he was informed about the ward and the behaviour of the staff. He was asked if he could intervene whilst retaining the anonymity of the former patients as this was a condition they had stipulated, not wanting to risk a backlash should they be admitted again. This he agreed to do and things seemed to change at least temporarily. This was, however, a very delicate matter because the future treatment of service users might have been at stake and we might have been deemed to have breached ethical procedures. Ethics is not a simple matter in research.

The second ethical dilemma was not nearly as serious but it was personally difficult and does have research implications. At the end of one focus group, a participant asked me if my experience had been the same as that described during the group. I had not anticipated this question although in hindsight it was eminently predictable given what we had disclosed. I did not give an adequate answer and mumbled something about it being more or less the same. This was a lesson for future research. We emphasized a principle of reciprocity at the start of the meeting and, at the end, violated it. Participatory research as practised in work such as this requires a balance between disclosure and not contaminating research. As the question came at the end of a first wave group, a detailed account of my own experience may have affected the nature of the second meeting. There might be a case for offering some of it at the end of people’s involvement in the work but we should not underestimate the emotional side of this [33]. The whole issue of ‘equality of participation’ when claims about advances in method rest on communality between researchers and participants requires much deeper interrogation. Certainly, there are more open ways of handling this in participatory research [34].

### Community validation

In terms of going outside the academy, we often work with a local user group to present and disseminate our work to find out what the local service user community reactions to it are. We took VOICE to the space where this group meet and held a workshop. Most liked the measure and one said that they wished it had been around when they were in inpatient settings. There were, however, two other reactions and they were very different. One person said they could never have completed the measure when in hospital because they had been so unwell. But a second described it as ‘simplistic’ and entailing a conception of service users as stupid. This gave us pause as we had always prized our measures as ‘easy to complete’. For the first time, a service user complained of our apparent perception of their and their community’s limited cognitive capacities and we have stopped being unwittingly patronizing. This has impacted how we now run feasibility studies, looking for ambiguity, certainly, but not over-simplifying.

### Modifying the model

Latterly, the model described above has changed and the views of professionals included [17, 35]. Indeed, they are included at the start, and therefore, in some sense frame the research. This is balanced by a consideration of service users’ views but not always by service user researchers [36]. Such a move departs from a wholly service-user developed model and it is an open question whether it is an improvement. The focus groups in Wykes et al. [22] were run by service user researchers but this was preceded by a Delphi exercise with professionals and followed by a very complex procedure borrowed from health economics and involving multiple statistical tests known as ‘forced choice experiments’. Such additions are intended to make the model more ‘scientific’ but one might also argue that it makes it less ‘pure’, certainly less user-centred. There is the potential for disagreement between user and conventional researchers and the criteria for which view is in the ascendant are not clear. It runs the additional risk of privileging the place of numbers above that of the views of service users. On the other hand, some might call this co-production.

### Is there a ‘community’ of mental health service users/survivors?

The answer to this question must be ‘no’ in any conventional sense. The vast literature on ‘stigma’ shows that probably the majority of people with a psychiatric diagnosis go to great lengths to hide it routinely [37]. However, neither is the situation quite that implied in the oft-quoted statistic that 1 in 4 people will suffer from a mental illness (https://www.time-to-change.org.uk/mental-health-statistics-facts). The implication is that these individuals are randomly distributed across the population; that mental illness can happen to ‘anyone’. It has been known for over half a century that inequalities are critical in determining who this ‘anyone’ might be [38], and sometimes attributes of marginalized groups are written into the very diagnostic categories themselves [39]. Yet anti-stigma campaigns often proceed in ignorance of such evidence. Further, despite isolation being a growing issue, mental health service users do often seek out each other for support, advocacy or campaigning activities. It did not surprise me that people in the focus groups knew each other well, and that those who did had experienced the most coercive practices. In England, day centres used to be places where service users could get together and often these were regarded as ‘safe’ places, as refuge from a hostile society [40]. Such spaces are now deemed a repository of ‘dependency’ and despite talk of social capital [41] and the importance of social networks for ‘recovery’ taught in ‘Recovery Colleges’ [42], the idea that these networks might be composed of other service users is frowned upon. Even peer support has lost its original meaning [43]. Indeed, recent initiatives seem designed to make the random ‘1 in 4’ picture a reality, to disrupt any connectivity between people similarly placed. And yet, there is resistance to this and people find in associating with each other not only a real sense of support but partake in forms of knowledge making that reimagine a different future where they become, sometimes for the first time, agents of collective change rather than suitable cases for treatment or something worse [44]. This does not entail a ‘community’ nor a homogenous or universal engagement with all designated mentally disordered. To claim such would be absurd. But it does mean that in unexpected places new resistances and resiliences are formed that do not take normative shapes. That these are fluid and often local is evident but in the small projects described here, we at least partially engaged with such voices. These associations can become more general through social media with closed group challenging mainstream ‘reforms’ in very novel and sophisticated ways. Examples are Recovery in the Bin and PD in the Bin. The names speak for themselves as long as one is aware of the play on words—the ‘Bin’ is slang for ‘mental hospital’ in this community and humour, often dark humour, is a hallmark of their non-normativity.

## Conclusion

The participatory research described here is on a small scale compared to the extension of the idea to international programmes and yet it is innovative, thorough and seems to work better at leveling power relations. Reflections show that some of the same problems pertain but, perhaps surprisingly, we are astute at overcoming or at least recognizing them. This, I argue, is because the position of user/survivor researcher is unique. We combine, rather synthesize, experiential and empirical and theoretic knowledge. Our specific model varies from most participatory research in the method used to turn measure generation on its head yet it retains a place in clinical research most relevant to social psychiatry. Above all, it foregrounds the voice of those usually positioned as merely subjects (read objects) of research without assuming that that ‘voice’ is unproblematic or not shaped by the research context itself as well as wider societal structures. The difficulties identified are not proposed as tensions to be ‘solved’ but as dilemmas that can be articulated so as better to facilitate good practice, not reach an unattainable perfect state.
